# Solubility measurement and thermodynamic modeling of *pantoprazole sodium sesquihydrate* in supercritical carbon dioxide

**DOI:** 10.1038/s41598-022-11887-1

**Published:** 2022-05-11

**Authors:** Gholamhossein Sodeifian, Chandrasekhar Garlapati, Fariba Razmimanesh, Hassan Nateghi

**Affiliations:** 1 Department of Chemical Engineering, Faculty of Engineering, University of Kashan, Kashan, 87317-53153 Iran; 2 Laboratory of Supercriritcal Fluids and Nanotechnology, University of Kashan, Kashan, 87317-53153 Iran; 3 Modeling and Simulation Centre, Faculty of Engineering, University of Kashan, Kashan, 87317-53153 Iran; 4 Department of Chemical Engineering, Puducherry Technological University, Puducherry, 605014 India

**Keywords:** Chemical engineering, Chemistry, Engineering

## Abstract

Knowing the solubility data of pharmaceutical compounds in supercritical carbon dioxide (ScCO_2_) is essential for nanoparticles formation by using supercritical technology. In this work, solubility of solid *pantoprazole sodium sesquihydrate* in ScCO_2_ is determined and reported at 308, 318, 328 and 338 K and at pressures between 12 and 27 MPa. The solubilities are ranged between 0.0301 $\times$ 10^–4^ and 0.463 $\times$ 10^–4^ in mole fraction. The determined solubilities are modelled with a new model using solid–liquid equilibrium criteria and the required activity coefficient is developed using regular solution theory. The measured solubilities data are also modelled with three recent and four conventional empirical models. The recent models used are, Alwi-Garlapati (AARD = 13.1%), Sodeifian et al. (14.7%), and Tippana-Garlapati (15.5%) models and the conventional models used are Chrastil (17.54%), reformulated Chrastil (16.30%), Bartle (14.1%) and Mendenz Santiago and Teja (MT) (14.9%) models. The proposed model is correlating the data with less than 14.9% and 16.23% in terms of AARD for temperature dependent and independent cases. Among exiting models, Mendez Santiago and Teja (MT) and Alwi-Garlapati models correlate the data better than other models (corresponding AARD% and AIC_c_ are 14.9, 13.1 and −518.89, −504.14, respectively). The correlation effectiveness of the models is evaluated in terms of Corrected Akaike’s Information Criterion (AIC_c_). Finally, enthalpy of solvation and vaporization of *pantoprazole sodium sesquihydrate* are calculated and reported. The new model proposed in this study can be used for the combination of any complex compound with any supercritical fluid.

## Introduction

The utilization of carbon dioxide (CO_2_) in its supercritical condition (commonly designated as ScCO_2_) in drug particle formation is evident in the literature^1–5^. The implementation of such supercritical technology needs an exact solubility data. The methods of measuring solubility data are well established in the literature and the data are usually available in a limited range^6–17^. Measuring solubility data at every condition would be cumbersome and appropriate modeling is required to address this task^18–20^. Solubility modeling is valuable and no single model would serve all the compounds, most of the times, the models are specific to compounds and due to this reason, numbers of models are developed to correlate the solubility data^20^. Exact solubilities measurements along with modeling are necessary for selecting the suitable particle micronization method using ScCO_2_. Further, it is observed in the literature that there is lack of information about the solubility data of many important drugs in ScCO_2_, therefore, the task of estimation of solubility of drugs in ScCO_2_ is imperative for the implementation of supercritical technology.

*Pantoprazole sodium sesquihydrate* is an important drug that is prescribed for the treatment of gastroesophageal reflux disease (GERD) and it proper dosage is critical in its treatment. Drug particle size greatly influences bioavailability of the drug which in turn influences the drug dosage. Currently, maximum of 20 mg per day of *pantoprazole sodium sesquihydrate* is being used for the treatment of gastroesophageal reflux disease^21^. Present study is helpful in the selection of a suitable method for the production of drug nanoparticles or microparticles by using supercritical technology, followed by a reduction in drug dosage. In order to pursue this, experimental solubility information of the drug is essential. However, the solubility of *pantoprazole sodium sesquihydrate* in ScCO_2_ was not reported in the literature, hence, measuring and modeling of its solubility are studied in this work. *Pantoprazole sodium sesquihydrate* is a typical compound as it has sodium in its structure and due to this, it is not possible to apply the group contribution methods to evaluate the critical properties and vapour pressure data. Thus, the equation of state (EoS) modeling is not applicable for the solubility data and there is need to develop a suitable solubility model to correlate the data. Therefore, in this work a new solubility model is proposed to correlate the solubility of *pantoprazole sodium sesquihydrate* in ScCO_2_. Further, models appeared in recent literature proposed by Alwi-Garlapati^22^, Sodeifian^23^ and Tippana-Garlapati^24^ as well as the conventional models proposed by Chrastil^25^, Reformulated Chrastil (R. Chrastil)^26^, Bartle^27^ and Mendez Santiago and Teja (MT)^28^ are explored. The conventional models (Reformulated Chrastil (R. Chrastil), Bartle) are mainly used to obtain necessary thermodynamic information of the solute from its solubility data. Mendez Santiago and Teja (MT) model is used to check its self-consistency. Alwi-Garlapati^22^ model is developed based on solid–liquid phase equilibrium criteria and Sodeifian and Tippana-Garlapati models are empirical models developed specifically for correlating solubility data of compounds in ScCO_2_. Finally, the correlating ability of different models is evaluated by Akaike’s Information Criterion (AIC_c_).

## Experimental section

### Chemical details

The CO_2_ and *Pantoprazole sodium sesquihydrate* were obtained from Fadak Company, Kashan (Iran). *Pantoprazole sodium sesquihydrate* was obtained from Temad Pharmaceutical Company, (Iran) (Table 1).

**Table 1:** Chemicals used in the work and its details.

Compound	Formula	Structure	M_W_ (g/mol)	T_m_ (K)	λ_max_ (nm)	CAS number	Minimum purity by supplier
*Pantoprazole sodium sesquihydrate*	C_16_H_14_F_2_N_3_NaO_4_S × 1.5 H_2_O		432.4	412	290	164579-32-2	99% (HPLC)
Carbon dioxide	CO_2_		44.01			124-38-9	99.99% (GC)
Deionized water	H_2_O		18.01				

### Experiment

**Figure 1:**
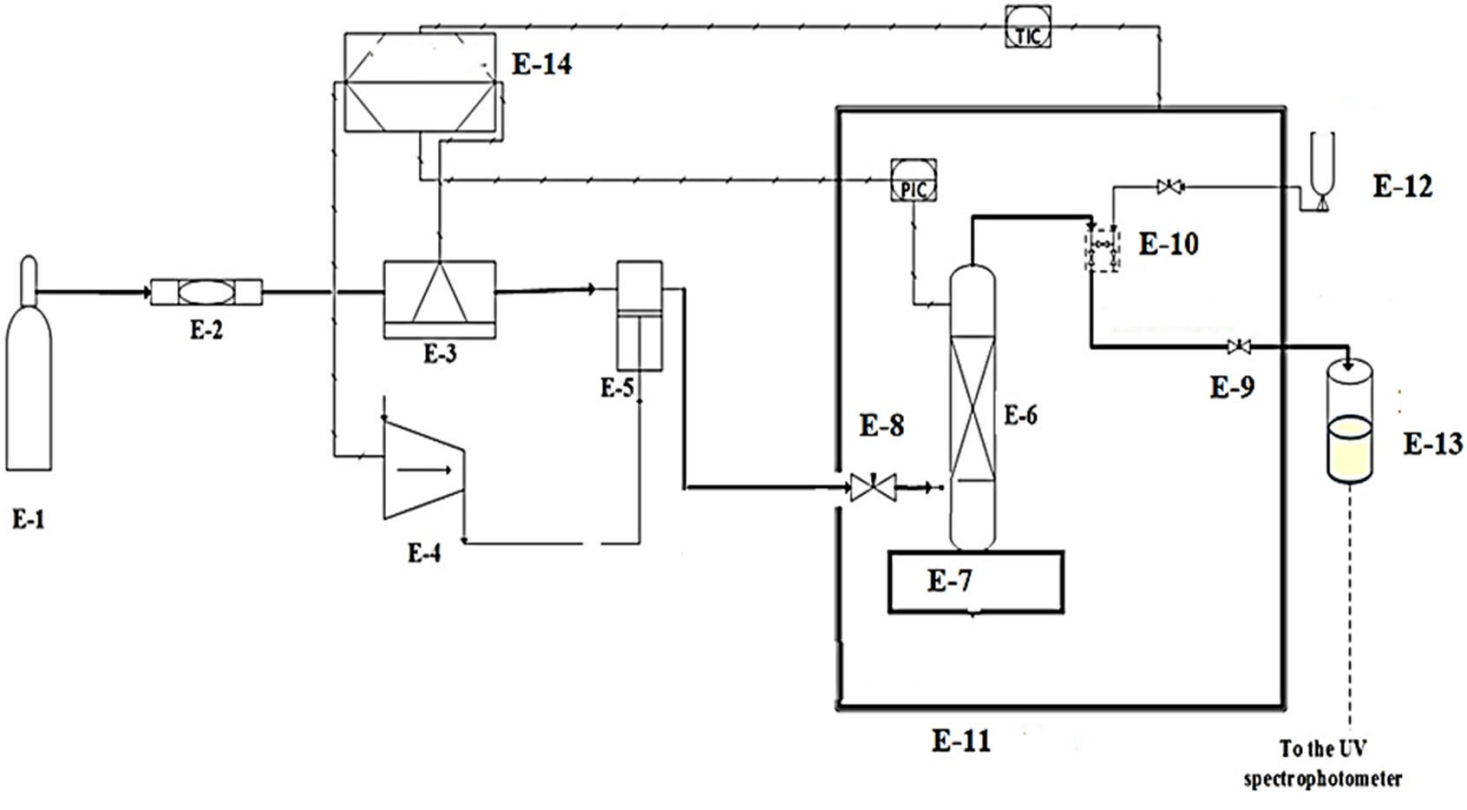
Device used for the measurement of solubility, E1 is the CO_2_ cylinder; E-2 is the Filter; E-3 is the Refrigerator unit; E-4 is the Air compressor; E-5 is the Pump; E-6 is the Equilibrium cell; E-7 is the Magnetic stirrer; E-8 is the Needle valve; E-9 is the Back-pressure valve; E-10is the Six-port valve; E-11 is the Oven; E-12 is the Syringe; E13 is the Collection vial; E-14 is the Control panel.

The equipment used for solubility measurement is shown in Fig. 1. The method utilized is considered as the isobaric-isothermal method^29^. Each measurement is performed with high precision, during experiments; temperature is maintained at desired value within ± 0.1 K. A known amount of *pantoprazole sodium sesquihydrate* drug (solute) has been used in the equilibrium cell to measure the solubility data. The capacity of the cell is 70 mL. A magnetic stirrer was mounted with the cell to measure the solubility data. A magnetic stirrer that is mounted with the equilibrium cell helps in attaining equilibrium between the solute and the ScCO_2_. To confirm equilibrium attainment, the experiments are done with a fresh sample at specified temperature and pressure at various time intervals (5 min, 10 min, 20 min, 30 min, 40 min, 50 min and 60 min) and the solubility readings are recorded. It is observed that the solubility is independent of time after 30 min. Thus, for correct results after 60 min, samples are considered for analysis. After equilibrium, 600 µL of a saturated sample is collected in dematerialized water (DM water’s conductivity is 1μS/cm) via a 6-way port, two-status valve. More details are readily available elsewhere^30,31^. This experimental setup has already been validated in the previous work with alpha-tocopherol and naphthalene^32^. Each experiment is carried out in triplicate.

Spectrophotometer (UV–Visible, Model UNICO-4802) is utilized to quantify the *pantoprazole sodium sesquihydrate* solubility. The drug test samples were prepared by dissolving known weights of drug in known volume of DM water. *Pantoprazole sodium sesquihydrate* samples were analyzed at 290 nm and calibrations curve was established, indicating R^2^ of 0.99.

The following sets of equations are used to calculate equilibrium mole fraction, *y*_2_, and solubility, *S* (g/L), in ScCO_2_:

1 $$y_{2} = \frac{{n_{solute} }}{{n_{solute} + n_{{CO_{2} }} }},$$

where:

2 $$n_{solute} = \frac{{C_{s} \left( \frac{g}{L} \right){\kern 1pt} V_{s} (L)}}{{M_{s} \left( \frac{g}{mol} \right)}},{\text{ and}}$$

3 $$n_{{CO_{2} }} = \frac{{V_{l} (L){\kern 1pt} \rho \left( \frac{g}{L} \right)}}{{M_{{CO_{2} }} \left( \frac{g}{mol} \right)}}$$

4 $$S\left( \frac{g}{L} \right) = \frac{{C_{s} \left( \frac{g}{L} \right){\kern 1pt} V_{s} (L)}}{{V_{l} (L)}}$$

where *n*_*solute*_ and *n*_*CO2*_ are moles of solute (*Pantoprazole sodium sesquihydrate*) and CO_2_ in the sampling loop, respectively, *C*_*s*_ is the solute concentration (g/L), *M*_*s*_ and *M*_*CO2*_ are molecular weights of the solute and CO_2_ and *S* (g/L) is the equilibrium solubility.

## Modeling

### New solution model

In this model, ScCO_2_ is treated as expanded liquid. At equilibrium, the fugacity of the solute in the solid phase is equal to that of liquid phase and the solubility can express as:

5 $$y_{2} = \frac{1}{{\gamma_{2}^{\infty } }}\frac{{f_{2}^{S} }}{{f_{2}^{L} }}$$

where $$\gamma_{2}^{\infty }$$ is activity coefficient of solute (drug) at infinitesimal dilution in solvent (ScCO_2_). The $${\raise0.7ex\hbox{${f_{2}^{S} }$} \!\mathord{\left/ {\vphantom {{f_{2}^{S} } {f_{2}^{L} }}}\right.\kern-\nulldelimiterspace} \!\lower0.7ex\hbox{${f_{2}^{L} }$}}$$ ratio is expressed as follows^33–35^,

6 $$\ln \left( {\frac{{f_{2}^{S} }}{{f_{2}^{L} }}} \right) = \frac{{\Delta H_{2}^{m} }}{RT}\left( {\frac{T}{{T_{m} }} - 1} \right) - \int\limits_{{T_{m} }}^{T} {\frac{1}{{RT^{2} }}} \left[ {\int\limits_{{T_{m} }}^{T} {\left[ {\Delta C_{p} } \right]dT} } \right]dT$$

where,$\Delta C_{p}$ is known as heat capacity difference of the solute in solid and liquid phases. For constant $\Delta C_{p}$, Eq. (6) reduced to Eq. (7).

7 $$\ln \left( {\frac{{f_{2}^{S} }}{{f_{2}^{L} }}} \right) = \frac{{\Delta H_{2}^{m} }}{RT}\left( {\frac{T}{{T_{m} }} - 1} \right) - \frac{{\Delta C_{P} }}{R}\left[ {\ln \left( {\frac{T}{{T_{m} }}} \right) - T_{m} \left( {\frac{1}{{T_{m} }} - \frac{1}{T}} \right)} \right]$$

Combining Eq. (7) with Eq. (5) gives the expression for the solubility model (Eq. (8)).

8 $$y_{2} = \frac{1}{{\gamma_{2}^{\infty } }}\exp \left[ {\frac{{\Delta H_{2}^{m} }}{RT}\left( {\frac{T}{{T_{m} }} - 1} \right) - \frac{{\Delta C_{p} }}{R}\left[ {\ln \left( {\frac{T}{{T_{m} }}} \right) - T_{m} \left( {\frac{1}{{T_{m} }} - \frac{1}{T}} \right)} \right]} \right]$$

In order to use Eq. (8), an appropriate model for $\gamma_{2}^{\infty }$ is needed.

In this work, the required $\gamma_{2}^{\infty }$ is obtained from regular solution theory and it is represented as Eq. (9)^36,37^.

9 $$\gamma_{2}^{\infty } = \exp \left[ {\frac{{V_{1} \varphi_{1}^{2} }}{RT}\left( {\delta_{2} - \delta_{1} } \right)^{2} } \right]$$

where $V_{1}$,$\varphi$, R, T, $\delta_{1}$ and $\delta_{2}$ are molar volume of ScCO_2_, volume fraction of ScCO_2_, universal gas constant, system temperature and solubility parameter of ScCO_2_ (solvent) and solubility parameter of drug (solute), respectively.

$\varphi$. $\delta_{1}$ and $\delta_{2}$ are mathematically represented as

10a $$\varphi = \frac{{x_{1} \rho_{1} }}{{x_{1} \rho_{2} + x_{2} \rho_{1} }}$$

10b $$\delta_{1} = \sqrt {a_{11} \rho_{1} }$$

10c $$\delta_{2} = \sqrt {a_{22} \rho_{2} }$$

Combining Eqs. (10a), (10b), (10c) with Eq. (9) and neglecting the term $x_{2} \rho_{1}$ in comparison to $x_{1} \rho_{2}$ gives Eq. (11) ^8^

11 $$\gamma_{2}^{\infty } = \exp \left[ {\frac{1}{RT}\left( {a_{22} + a_{11} \frac{{\rho_{1} }}{{\rho_{2} }} - 2\sqrt {a_{11} a_{22} } \left( {\frac{{\rho_{1} }}{{\rho_{2} }}} \right)^{0.5} } \right)} \right]$$

Equation (11) is further reduced in terms of molar volume of solute ($v_{2}$) as Eq. (12)

12 $$\gamma_{2}^{\infty } = \exp \left[ {\frac{1}{RT}\left( {a_{22} + a_{11} v_{2} \rho_{1} - 2\sqrt {a_{11} a_{22} } \left( {v_{2} \rho_{1} } \right)^{0.5} } \right)} \right]$$

Combining Eq. (12) with Eq. (9) gives a new explicit solubility model, (Eq. (13))

13 $$y_{2} = {{\exp \left[ {\frac{{\Delta H_{2}^{m} }}{RT}\left( {\frac{T}{{T_{m} }} - 1} \right) - \frac{{\Delta C_{p} }}{R}\left[ {\ln \left( {\frac{T}{{T_{m} }}} \right) - T_{m} \left( {\frac{1}{{T_{m} }} - \frac{1}{T}} \right)} \right]} \right]} \mathord{\left/ {\vphantom {{\exp \left[ {\frac{{\Delta H_{2}^{m} }}{RT}\left( {\frac{T}{{T_{m} }} - 1} \right) - \frac{{\Delta C_{p} }}{R}\left[ {\ln \left( {\frac{T}{{T_{m} }}} \right) - T_{m} \left( {\frac{1}{{T_{m} }} - \frac{1}{T}} \right)} \right]} \right]} {\exp \left[ {\frac{1}{RT}\left( {a_{22} + a_{11} v_{2} \rho_{1} - 2\sqrt {a_{11} a_{22} } \left( {v_{2} \rho_{1} } \right)^{0.5} } \right)} \right]}}} \right. \kern-\nulldelimiterspace} {\exp \left[ {\frac{1}{RT}\left( {a_{22} + a_{11} v_{2} \rho_{1} - 2\sqrt {a_{11} a_{22} } \left( {v_{2} \rho_{1} } \right)^{0.5} } \right)} \right]}}$$

Equation (13) indicates that solubility is a function of several quantities, which are melting enthalpy of the solute ($\Delta H_{2}^{m}$), melting temperature of the solute ($T_{m}$), heat capacity difference of solute between solid and expanded liquid phases ($\Delta C_{p}$), temperature (T), molar volume of the solute ($v_{2}$), ScCO_2_ density ($\rho_{1}$), interaction potential of the solvent–solvent molecule ($a_{11}$) and interaction potential of the solute–solute molecule ($a_{22}$). In this model, it is assumed that $\Delta H_{2}^{m}$,$T_{m}$, $v_{2}$ and $\rho_{1}$ are known/fixed. Therefore, for an isotherm (i.e., known T),$\Delta C_{p}$, $a_{11}$ and $a_{22}$ are adjustable parameters; further, over a small temperature range these parameters may be treated as constants. In the case of unavailability of experimental data of $\Delta H_{2}^{m}$, $T_{m}$ and $v_{2}$ are estimated with the help of suitable group contribution method. Sometimes, presence of sodium like metals in solute compounds hinders the applicability of group contribution method to evaluate the melting enthalpy and activity coefficient. In such cases, the term $6.54\left( {1 - {{T_{m} } \mathord{\left/ {\vphantom {{T_{m} } T}} \right. \kern-\nulldelimiterspace} T}} \right)$ is used in place of term ${{\Delta H_{2}^{m} } \mathord{\left/ {\vphantom {{\Delta H_{2}^{m} } {RT\left( {{T \mathord{\left/ {\vphantom {T {T_{m} - 1}}} \right. \kern-\nulldelimiterspace} {T_{m} - 1}}} \right)}}} \right. \kern-\nulldelimiterspace} {RT\left( {{T \mathord{\left/ {\vphantom {T {T_{m} - 1}}} \right. \kern-\nulldelimiterspace} {T_{m} - 1}}} \right)}}$ ^36,38^. Thus, the final expression for the solubility becomes Eq. (14).

14 $$y_{2} = {{\exp \left[ {6.54\left( {1 - \frac{T}{{T_{m} }}} \right) - \frac{{\Delta C_{p} }}{R}\left[ {\ln \left( {\frac{T}{{T_{m} }}} \right) - T_{m} \left( {\frac{1}{{T_{m} }} - \frac{1}{T}} \right)} \right]} \right]} \mathord{\left/ {\vphantom {{\exp \left[ {6.54\left( {1 - \frac{T}{{T_{m} }}} \right) - \frac{{\Delta C_{p} }}{R}\left[ {\ln \left( {\frac{T}{{T_{m} }}} \right) - T_{m} \left( {\frac{1}{{T_{m} }} - \frac{1}{T}} \right)} \right]} \right]} {\exp \left[ {\frac{1}{RT}\left( {a_{22} + a_{11} v_{2} \rho_{1} - 2\sqrt {a_{11} a_{22} } \left( {v_{2} \rho_{1} } \right)^{0.5} } \right)} \right]}}} \right. \kern-\nulldelimiterspace} {\exp \left[ {\frac{1}{RT}\left( {a_{22} + a_{11} v_{2} \rho_{1} - 2\sqrt {a_{11} a_{22} } \left( {v_{2} \rho_{1} } \right)^{0.5} } \right)} \right]}}$$

In Eq. (14), $\Delta C_{p}$, $a_{11}$ and $a_{22}$ are adjustable constants and thus it is a three parameters model. It is very important to note that proposed solution model essentially requires the solute’s physical property (i.e., melting temperature) and density of *ScCO*_*2*_.Therefore, the new model proposed in this study cannot be applied to the system whose melting point is not known.

From the literature, it is clear that the solubility is highly a nonlinear function of density, pressure and temperature^24^. The ability of a particular model in correlating the solubility data is also not clear due to its nonlinearity, so, several models are used for the correlation purpose. The models used are few latest models and conventional models. The other purpose of the conventional models is to estimate the essential thermodynamic information such as total heat, sublimation and solvation enthalpies. More details of the same are presented in the following section.

### Recent models

#### Alwi-Garlapati model

It is a simple model and its basis is thermodynamic frame work. According to the model, at equilibrium, solute’s chemical potentials in both solid and liquid phases are equal. Further, solid sublimation pressure is assumed to obey Antoine’s equation and sublimation pressure to temperature ratio is negligible when it is compared to total pressure to temperature ratio. Thus, the final expression for the solubility ($y_{2}$) in terms of reduced density (i.e.,$\rho_{1r} = {{\rho_{1} } \mathord{\left/ {\vphantom {{\rho_{1} } {\rho_{c} }}} \right. \kern-\nulldelimiterspace} {\rho_{c} }}$) and reduced temperature (i.e.,$T_{r} = {T \mathord{\left/ {\vphantom {T {T_{c} }}} \right. \kern-\nulldelimiterspace} {T_{c} }}$) is:

15 $$y_{2} = \frac{1}{{\rho_{r} T_{r} }}\exp \left( {A_{0} + \frac{{A_{1} }}{{T_{r} }} + A_{2} \rho_{1r} } \right)$$

where $A_{0} - A_{2}$ are model constants.

#### Sodeifian et al., model

It is a highly nonlinear mathematical model and correlates solubility in terms of pressure, temperature and density as:

16 $$\ln \left( {y_{2} } \right) = B_{0} + \frac{{B_{1} P^{2} }}{T} + B_{2} \ln \left( {\rho_{1} T} \right) + B_{3} \left( {\rho_{1} \ln \left( {\rho_{1} } \right)} \right) + B_{4} P\ln \left( T \right) + B_{5} \frac{{\ln \left( {\rho_{1} } \right)}}{T}$$

where $B_{0} - B_{5}$ are model constants.

#### Reddy-Garlapati model

It is a dimensionless empirical model and correlates solubility in terms of reduced pressure and reduced temperature as:

17 $$y_{2} = \left( {D_{0} + D_{1} P_{r} + D_{2} P_{r}^{2} } \right)T_{r}^{2} + (D_{3} + D_{4} P_{r} + D_{5} P_{r}^{2} )$$

where $D_{0} - D_{5}$ are model constants.

### Conventional models

#### Chrastil model

It is the first solvate complex model and correlates solubility as a function of supercritical fluid density and temperature as:

18 $$y_{2} = \frac{{\left( {\rho_{1} } \right)^{\kappa - 1} \exp \left( {E_{0} + \frac{{E_{1} }}{T}} \right)}}{{\left[ {1 + \left( {\rho_{1} } \right)^{\kappa - 1} \exp \left( {E_{0} + \frac{{E_{1} }}{T}} \right)} \right]}}$$

where $\kappa$ and $E_{0} - E_{1}$ are model constants.

Since Eq. (13) is dimensionally inconsistent^24,26,39^, it is dimensionally corrected and known as *Reformulated Chrastil model:*

19 $$y_{2} = \left( {\frac{{RT\rho_{1} }}{{M_{scf} f^{ \bullet } }}} \right)^{{\kappa^{\prime} - 1}} \exp \left( {F_{0} + \frac{{F_{1} }}{T}} \right)$$

where $\kappa^{\prime}$ and $E_{0} - E_{1}$ are model constants.

#### Bartle et al., model

It is one of the successful empirical models and correlates solubility as a function of temperature, supercritical fluid density and total pressure as:

20 $$\ln \left( {\frac{{y_{2} P}}{{P_{ref} }}} \right) = G_{0} + \frac{{G_{1} }}{T} + G_{2} \left( {\rho_{1} - \rho_{ref} } \right)$$

where $G_{0} - G_{2}$ are model constants. From parameter $G_{1}$, the vaporization enthalpy is $\Delta_{vap} H = - G_{1} R$ in which *R* is universal gas constant.

### Mendez Santiago and Teja (MT) model

It is conceptually developed on the statement of enhancement factor. According to this model, solubility is a function temperature, pressure and supercritical fluid density:

21 $$T\ln \left( {y_{2} P} \right) - H_{2} T = H_{0} + H_{1} \rho_{1}$$

When solubility data is casted on a plot as “$T\ln \left( {y_{2} P} \right) - H_{2} T$ vs. $\rho_{1}$”, all experimental data points irrespective of temperature collapse on to a single line (which is obtained out of calculated data). This model is usually used to check the generated data’s self-consistency.

## Results and discussion

**Figure 2:**
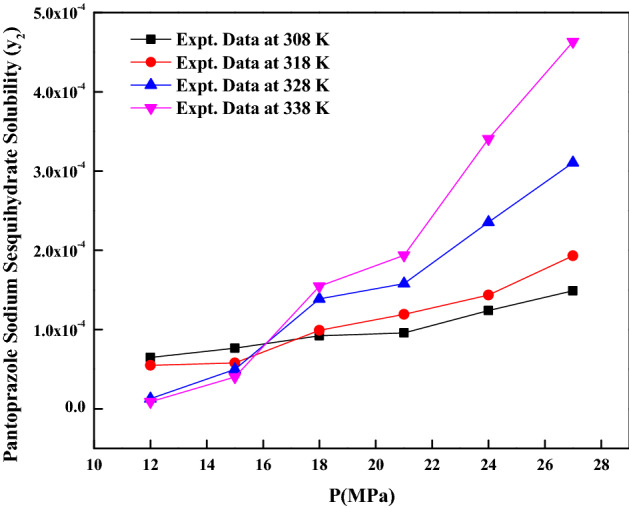
*Pantoprazole sodium sesquihydrate* solubility vs. pressure.

**Figure 3:**
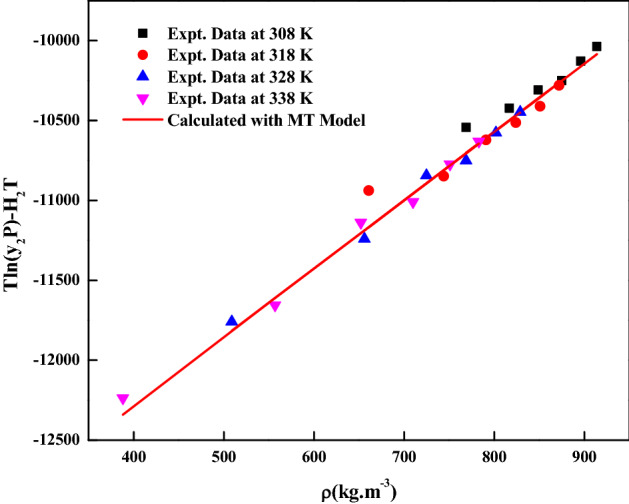
Solubility data self-consistency plot based on MT model.

The *pantoprazole sodium sesquihydrate* solubility in ScCO_2_ is determined at 308, 318, 328 and 338 K and at pressures between 12 and 27 MPa. The measured data is reported in Table 2. The reported ScCO_2_ densities are obtained from standard literature^40^. The high operating pressure increases solvent’s density and reduces intermolecular spaces between carbon dioxide molecules which increase interactions between the drug and ScCO_2_ molecules and thus causes an enhancement of ScCO_2_’s solvating power. Also, *pantoprazole sodium sesquihydrate*’s solubility is influenced by the complex effect of operating temperature which has a simultaneous effect on solute’s sublimation pressure, solvent density and obviously intermolecular interactions in the supercritical fluid phase^12,41,42^. From Fig. 2, it is observed that cross over pressure is around 16.0 MPa, further, solubility decreases with increasing temperatures and increases with increasing temperature below and above cross over pressure. The self-consistency is indicated in the Fig. 3. From this figure, it is observed that all measured data fall into a line which indicates that the solubility data in this work is self-consistent.

**Table 2:** Solubility of pantoprazole sodium sesquihydrate in ScCO_2_at various temperatures and pressures.

Temperature (K)^a^	Pressure (MPa)^a^	Density of ScCO_2_ (kg/m^3^)^40^	y_2_ × 10^4^ (mole fraction)	Experimental standard deviation, S(ȳ) × (10^4^)	S (equilibrium solubility) (g/L)	Expanded uncertainty of mole fraction (10^4^ U)
308	12	769	0.0648	0.001	0.0435	0.0036
	15	817	0.0764	0.003	0.0544	0.0069
	18	849	0.0921	0.004	0.0682	0.0090
	21	875	0.0958	0.004	0.0731	0.0091
	24	896	0.1239	0.006	0.0968	0.0132
	27	914	0.1489	0.006	0.1183	0.0137
318	12	661	0.0548	0.002	0.0316	0.0047
	15	744	0.0580	0.002	0.0377	0.0048
	18	791	0.0990	0.004	0.0682	0.0091
	21	824	0.1192	0.003	0.0856	0.0080
	24	851	0.1436	0.004	0.1064	0.0102
	27	872	0.1930	0.007	0.1467	0.0164
328	12	509	0.0381	0.001	0.0170	0.0026
	15	656	0.0498	0.001	0.0285	0.0030
	18	725	0.1388	0.003	0.0877	0.0086
	21	769	0.1579	0.004	0.1059	0.0106
	24	802	0.2354	0.003	0.1646	0.0120
	27	829	0.3106	0.005	0.2243	0.0170
338	12	388	0.0301	0.001	0.0101	0.0024
	15	557	0.0403	0.002	0.0196	0.0044
	18	652	0.1548	0.002	0.0880	0.0080
	21	710	0.1938	0.004	0.1200	0.0118
	24	751	0.3408	0.006	0.2231	0.0192
	27	783	0.4634	0.003	0.3163	0.0213

The experimental standard deviation was obtained by $S\left( {y_{k} } \right) = \sqrt {\frac{{\sum\limits_{{}}^{{}} {\left( {y_{j} - \overline{y}} \right)^{2} } }}{n - 1}}$. Expanded uncertainty (U) = k*u_*combined*_ and the relative combined standard uncertainty *u*_*combined*_*/y* = $\sqrt {\sum\limits_{i = 1}^{N} {(P_{i} {\kern 1pt} u(x_{i} )/x_{i} )^{2} } }$.

^a^Standard uncertainty u are u(T) =  ± 0.1 K; u(p) =  ± 0.1 MPa. Also, relative standard uncertainties are obtained below 5% for mole fractions and solubilities. The value of the coverage factor k = 2 was chosen on the basis of the level of confidence of approximately 95 percent.

**Figure 4:**
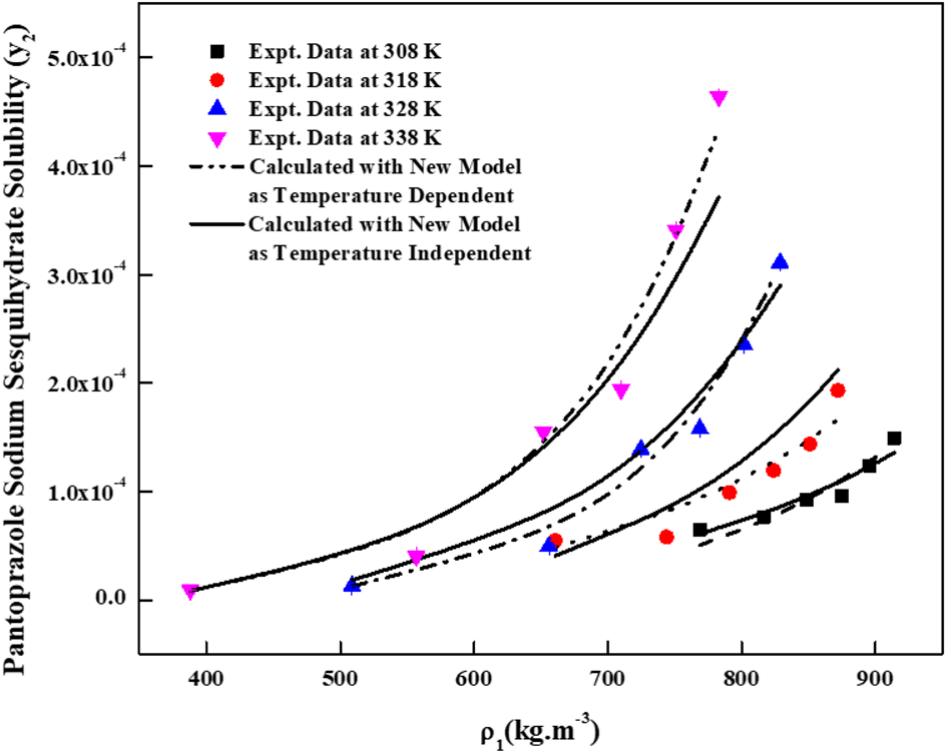
*Pantoprazole sodium sesquihydrate* solubility vs.$\rho_{1}$. Lines are new model calculations as temperature independent; dash, dot, dash dot and dash dot dot lines are new model calculations as temperature dependent.

**Figure 5:**
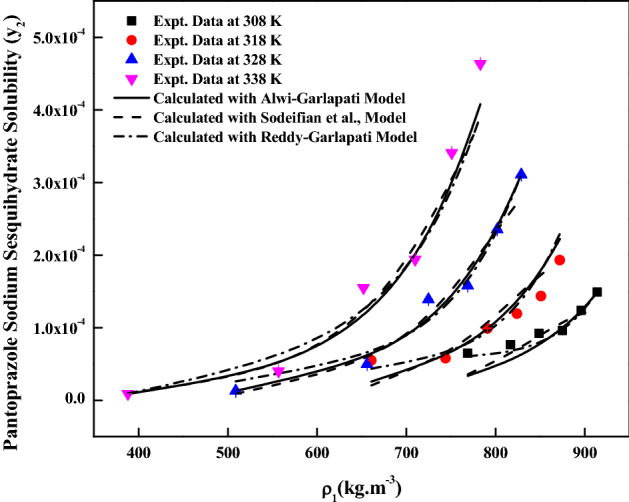
*Pantoprazole sodium sesquihydrate* solubility vs. $\rho_{1}$. Lines are Alwi-Garlapati model calculations; dashed lines are Sodeifian et al., model calculations; dash dot lines are Reddy-Garlapati model calculations.

**Figure 6:**
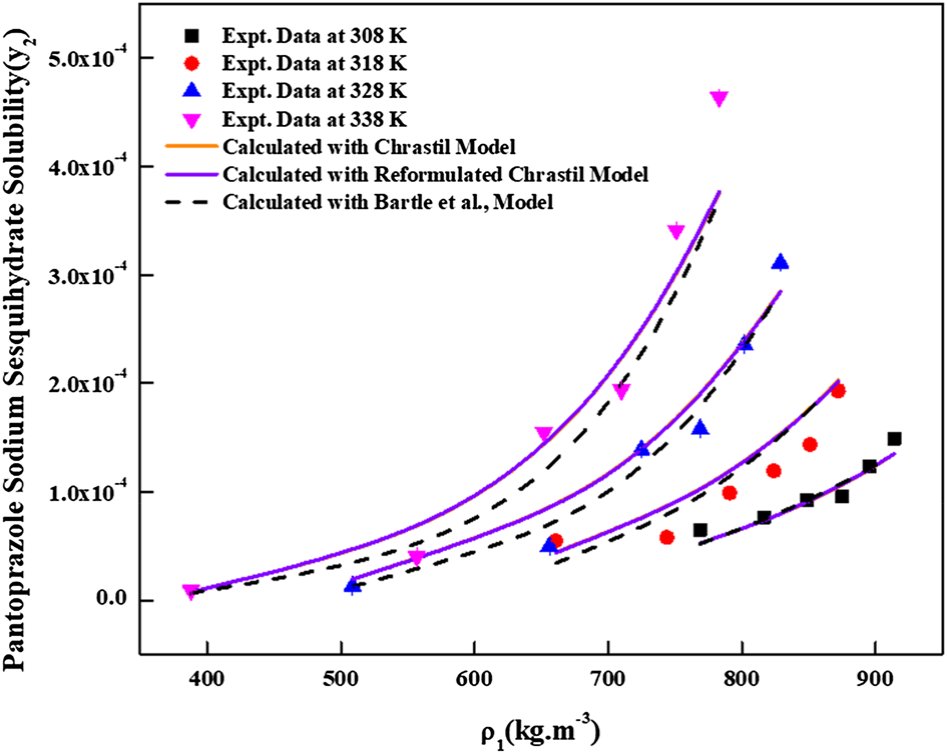
*Pantoprazole sodium sesquihydrate* solubility vs.$\rho_{1}$. Lines are Chrastil and Reformulated Chrastil model calculations; dashed lines are Bartle et al., model calculations.

The new solution model proposed in this work has three adjustable parameters ($\Delta C_{p}$, $a_{11}$ and $a_{22}$). For regression, these parameters are treated as temperature dependent and temperature independent. Although conceptually, these parameters are temperature dependent^43,44^**,** however, in literature, these parameters are handled as temperature independent over a small temperature range^45^. Therefore, both temperature dependent and independent results are reported in this study. For regression, melting temperature and molar volume of *pantoprazole sodium sesquihydrate* are needed. The required melting temperature is obtained from the material’s source and the molar volume of the solid *pantoprazole sodium sesquihydrate* is calculated using Immirzi and Perini method^36^. The material safety data indicates that the melting temperature is 412 K and calculated molar volume is 2.8202 × 10^–4^ m^3^/mol. The proposed model correlates the data less than 14.9% and 16.23% in terms of AARD% for temperature dependent and independent cases, respectively. Table 3 shows all the new model correlations. The correlating ability of the new model proposed in this study is indicated in Fig. 4. The correlations of the solubility data with temperature dependent parameters are better than temperature independent parameters. Alwi-Garlapati, Sodeifian et al., and Reddy-Garlapati models correlate the solubility data. The correlations constants are reported in Table 4. The regression ability of the recent models for the solubility prediction is indicated in the Fig. 5. The correlations of the data are quite satisfactory for Alwi-Garlapati model compared to Reddy-Garlapati and Sodeifian models. The correlation constants of conventional models as temperature independent are reported in Table 5. The correlating ability of the recent models is indicated in Fig. 6. From the conventional model constants, the thermodynamic properties, namely total heat of enthalpy of vaporization and solvation are calculated and reported in Table 6. The vaporization enthalpy obtained for Bartle et al., model is 72.18 kJ/mol. From Chrastil model, total heat is −59.432 kJ/mol (i.e., −7147.4 × R, where R is universal gas constant). Solvation enthalpy is obtained from the difference between total and vaporization enthalpies. Solvation enthalpy for Bartle et al., model and Chrastil model combination is −15.829 kJ/mol and the negative sign is attributed since the solvation enthalpy is exothermic. Similarly, from the reformulated Chrastil and Bartle et al., models combination, solvation enthalpy is −35.996 kJ/mol.

**Table 3:** Correlation constants of the new model.

New model, eq	Temperature, K	Correlation parameters	AARD%	R^2^
As temperature dependent	308	$a_{11}$ = 1.0939 × 10^6^ $a_{22}$ = 1.3423 × 10^3^ $\Delta C_{p}$ = −8.7915 × 10^3^	6.40	0.917
	318	$a_{11}$ = 1.7124 × 10^6^ $a_{22}$ = 1.1794 × 10^2^ $\Delta C_{p}$ = −1.7208 × 10^4^	11.4	0.928
	328	$a_{11}$ = 1.9675 × 10^6^ $a_{22}$ = 2.267 × 10^3^ $\Delta C_{p}$ = −2.4926 × 10^4^	9.28	0.983
	338	$a_{11}$ = 2.0213 × 10^6^ $a_{22}$ = 2.0409 × 10^3^ $\Delta C_{p}$ = −3.3373 × 10^4^	14.9	0.985
As temperature independent	308–338	$a_{11}$ = 6.6074 × 10^4^ $a_{22}$ = 1.7996 × 10^5^ $\Delta C_{p}$ = 21.618	16.23	0.944

**Table 4:** Correlation constants of the recent models.

Model	Correlation parameters	AARD%	R^2^	R^2^_adj_
Alwi-Garlapati model	*A*_0_ = 9.0006 *A*_1_ = −28.013 *A*_2_ = 5.3824	13.1	0.957	0.950
Sodeifian et al., model	*B*_0_ = −12.725 *B*_1_ = −2.874 × 10^–3^ *B*_2_ = 3.1435 *B*_3_ = 1.3706 × 10^–3^ *B*_4_ = −0.02141 *B*_5_ = −2201.2	14.7	0.953	0.937
Reddy and Garlapati model	*D*_0_ = −1.2535 × 10^–3^ *D*_1_ = 5.5793 × 10^–6^; D_2_ = 2.7731 × 10^–4^ *D*_3_ = 1.3763 × 10^–3^; *D*_4_ = -5.7085 × 10^–5^ *D*_5_ = −2.6286 × 10^–4^	15.5	0.958	0.943

**Table 5:** Correlation constants of the conventional models.

Model	Correlation parameters	AARD%	R^2^	R^2^_adj_
Chrastil model	$\kappa$ = 7.3712 *E*_0_ = −29.074 *E*_1_ = −7147.4	17.54	0.933	0.923
Reformulated Charstil model	$\kappa^{\prime}$ = 6.9821 *F*_0_ = −58.493 *F*_1_ = −4791	16.30	0.955	0.948
Bartle et al., model	*G*_0_ = 23.454 *G*_1_ = −9052.4 *G*_2_ = 1.226 × 10^–2^	14.10	0.950	0.942
Mendenz Santiago and Teja model	H_0_ = −13,995 *H*_1_ = 4.2779 *H*_2_ = 29.372	14.90	0.975	0.918

**Table 6:** Calculated thermodynamic properties of pantoprazole sodium sesquihydrate.

Model	Thermodynamic quantity		
	Total enthalpy, ΔH_total_ (kJ/mol)	Enthalpy of vaporization ΔH_vap_ (kJ/mol)	Enthalpy of solvation,$\Delta H_{sol}$ (kJ/mol)
Chrastil model	59.432^a^		−15.829^d^
Reformulated Chrastil model	39.832^b^		−35.429^e^
Bartle et al., model		75.261^c^ (approximate value)	

^d^Magnitude difference between the ΔH_vap_^c^ and ΔH_total_^a^.

^e^Magnitude difference between the ΔH_vap_^c^ and ΔH_total_^b^.

Statistical comparisons of various models are conveniently carried out with Corrected AICc criterion^38–41^. Mathematically, AIC_c_ is represented as:

22 $$AI{C}_{c}=n\hspace{0.33em}\mathit{ln}\left({\sigma }^{2}\right)+2Q+\frac{2Q\left(Q+1\right)}{n-Q-1}$$

In Eq. (22), σ, n and $Q$ are variance of deviations, number of experimental data points and number of constants in a particular model, respectively. Table 7 indicates calculated AICc values. From the magnitude of AIC_c_, one can conclude the correlating efficacy of the models and the best model has the least value. From AIC_c_ information of various models, MT and Alwi-Garlapati models are able to correlate the data better than the other models. The new model when treated as temperature independent, it correlates the data on par with Sodeifian et al. and Chrastil models.

**Table 7:** Computed SSE, RMSE andAIC_c_values for various models.

Model	SSE $\times$ 10^8^	RMSE $\times$ 10^5^	n	K	AIC_c_
**New model**					
As temperature independent	2.65974	3.329	24	3	−487.69
**Recent models**					
Alwi-Garlapati model	1.34046	2.36331	24	3	−504.14
Sodeifian et al., model	1.60651	2.58724	24	6	−490.05
Reddy- Garlapati model,	1.43877	2.44844	24	6	−492.70
**Conventional models**					
Chrastilmodel	3.56118	3.852	24	3	−480.69
R. Chrastilmodel	2.1846	3.017	24	3	−492.42
Bartle model	1.92404	2.8314	24	3	−495.46
MT model	72.5	8.51	24	3	−518.89

## Conclusion

*Pantoprazole sodium sesquihydrate’s* solubility in ScCO_2_ is reported at 308, 318, 328, and 338 K in the pressure range of 12–27 MPa, for the first time. The solubilities were ranged between 0.0301 $\times$ 10^–4^ and 0.463 $\times$ 10^–4^ in mole fraction. For modeling, three recently developed solubility models and four conventional empirical solubility models were used. Further, measured data has been used to develop a new solubility model. Among various models, Alwi-Garlapati model is observed to correlate the data with the least AARD (13.1%). The correlating ability of various equations have been observed in terms of AIC_c_ values (ascending) as follows: MT (−518.89), Alwi-Garlapati (−504.14), Bartle (−495.46), Reddy and Garlapati (−492.70), R. Chrastil (−492.42), Sodeifian et al. (490.05), models, new model as temperature independent (−487.69) and Chrastil model (−480.69). The new model proposed in this study can be used for the combination of any complex compound with any supercritical fluid.

## Data Availability

All data generated or analyzed during this study are included in this published article.

## List of symbols

$$A_{0} - A_{2}$$: Eq. (15) parameters
AARD%: Average absolute relative deviation percentage
AIC: Akaike’s information criterion
AIC_c_: Corrected AIC
Adj.R^2^: Statistical parameter
*B*_0_–*B*_5_: Eq. (16) parameters
*C*_p_: Heat capacity
*D*_0_–*D*_5_: Eq. (17) parameters
*E*_*0*_*–E*_*2*_: Eq. (18) parameters
$f^{ \bullet }$: Standard state [fugacity(1 bar)] in Eq. (18)
*F*_0_–*F*_2_: Eq. (19) parameters
*G*_*0*_*–G*_*2*_: Eq. (20) parameters
H_sol_, H_sub_ & H_total_: Enthalpy (KJ/mol)
*H*_0_–*H*_2_: Eq. (21) parameters
M_scf_: Solvent molecular mass
n: Number of data points in Eq. (22)
*P*: Pressure (bar or MPa)
Q: Number of parameters of a model in Eq. (22)
*R*: Gas constant, J/(mol K)
*R*^2^: Square of coefficient of regression
RMSE: Root mean square error
SSE: Sum of squares due to error
T: Temperature, K
*V*_s_: Molar volume of solid solute, m^3^/mol
$y_{1}$: Solvent mole fraction
$y_{2}$: Solute mole fraction

## Greek symbols

$\kappa$: Eq. (19) constant (association number)
$\kappa^{\prime}$: Eq. (20) constant (association number)
$\rho_{1}$: Solvent density (kg/m^3^)
σ: Variance of deviations in Eq. (22)
Δ: Difference

## Subscripts

c: Critical
cal: Calculated
exp: Experimental
*ScCO*_2_: Supercritical carbon dioxide
sol: Solvation
sub: Sublimation
r: Reduced
ref: Reference state
total: Total
1: Solvent (ScCO_2_)
2: Solute
